# Beyond the platform: why surgical quality and surgeon proficiency outweigh the RATS vs. VATS debate in early-stage lung cancer

**DOI:** 10.3389/fonc.2026.1896248

**Published:** 2026-07-15

**Authors:** Marcello Migliore, Kwon Joong Na

**Affiliations:** 1 Thoracic Surgery and Transplantation, The Lung Health Centre, Organ Centre of Excellence (OCoE), King Faisal Specialist Hospital & Research Centre, Riyad, Saudi Arabia; 2 Medical College, Al Faisal University, Riyadh, Saudi Arabia; 3 Department of Surgery and Medical Specialties, University of Catania, Catania, Italy; 4 Department of Thoracic and Cardiovascular Surgery, Seoul National University Hospital, Seoul, Republic of Korea; 5 Cancer Research Institute, Seoul National University College of Medicine, Seoul, Republic of Korea

**Keywords:** humanoid surgeon, minimally invasive surgery, RATS, robotic surgery, surgical platform, VATS

## Abstract

The debate regarding the oncological superiority of Robotic-Assisted Thoracic Surgery (RATS) versus Video-Assisted Thoracic Surgery (VATS) for early-stage lung cancer is ongoing. Current evidence suggests comparable oncological outcomes between the two approaches. This perspective posits that long-term survival relies less on the platform itself and more on the “surgeon factor”—specifically, the tenacity for radical lymph node dissection. While RATS imposes higher costs, its technical advantages may lower the barrier to achieving standardized, high-quality dissection, effectively “democratizing” surgical precision. Consequently, the choice of approach should not be binary but tailored to anatomical complexity and surgeon proficiency. Rather than forcing a transition, future paradigms must focus on leveraging technology to minimize performance variability while maintaining cost-effectiveness in a value-based healthcare system.

## Introduction

Twenty years ago, the landscape of thoracic surgery was dominated by Video-Assisted Thoracic Surgery (VATS). At that time, Robotic-Assisted Thoracic Surgery (RATS) was in its infancy a novelty introduced in select centers but rarely considered a standard of care. Surgeons were rigorously rooted in VATS, and the community witnessed firsthand how it revolutionized patient recovery compared to thoracotomy. However, the surgical landscape began to shift rapidly. We all witness that recently academic conferences are dominated by the versatility of robotic platforms, and patients increasingly arrive well-informed, explicitly asking whether the “latest technology” equates to a cure.

This rapid paradigm shift forces us to ask a fundamental question: Does the platform itself truly dictate the oncological outcome? While the technological allure of RATS is undeniable, current evidence suggests that the platform is not the sole determinant of survival.

## Oncologic outcomes

Over the past decade, numerous studies have compared the outcomes of RATS and VATS. The consensus is largely one of equivalence (1–3). For instance, the recent multicenter randomized controlled trial (RVlob Trial) confirmed that RATS is non-inferior to VATS in terms of perioperative outcomes and oncological efficacy for resectable non-small-cell lung cancer (4). This consensus is further supported by institutional series. Park et al. compared early outcomes of robotic versus video-assisted thoracoscopic anatomical resection and demonstrated that complete resection rate (VATS 97.8% vs. RATS 98.5%, p=1.000) and postoperative complication rate (VATS 17.5% vs. RATS 19.0%, p=0.874) were not significantly different between VATS and RATS. They concluded that RATS is a safe and feasible procedure with perioperative results comparable to those of VATS (5–8).

This reinforces the notion that the biological behavior of the tumor and the completeness of the resection are far more impactful than the specific minimally invasive modality employed. The platform serves as the tool, not the defining factor. Yet, the real question is: who wields the tool?

## Lymphadenectomy

If the platform is not the primary driver of survival, what is? Lerut T. persuasively stated in his seminal editorial, “The surgeon as a prognostic factor” (9), the technical precision, judgment, and “determination” of the surgeon to perform a radical hilar and mediastinal lymph node dissection are paramount. A complete resection (R0) with adequate lymphadenectomy is the cornerstone of lung cancer surgery, and this depends entirely on the operator’s will and skill. While some studies report similar nodal upstaging rates between platforms (13), others, such as Wilson et al., have demonstrated that RATS yields significantly higher nodal upstaging rates compared to VATS (10.9% vs 8.1%), comparable to those of thoracotomy (11, 12). This is in contrast with those who believe that the determination to remove more lymph node is correlated with specialist interest in the pathology (9). For example, surgeons with no specialist interest were more likely (38%) to sample three or fewer axillary lymph nodes per patient than specialist breast surgeons (10%). Furthermore, even a surgeon’s case volume strongly influences operative outcomes, emphasizing the importance of proficiency and experience (10).

## Learning curve

In the VATS era, the steep learning curve could create a disparity in surgical quality between high-volume “master” surgeons and those with less experience (11). Nevertheless, the metric of interest and the technical competency with VATS influenced the learning curve for RATS lobectomy which was identified to be 25.3 ± 12.6 cases (14). Furthermore, when surgeons were considered experts in VATS technical proficiency was achieved earlier in the learning process (11).

Herein lies the true value of the robotic platform. It is not that RATS inherently could create better survival, but that it may facilitate excellence in surgery. Statements suggesting that RATS may “standardize excellence” or reduce surgeon-related variability should be moderated, as these remain attractive but not yet objectively proven concepts.

Nevertheless, it is known that the robotic platform could lower the technical barrier to complex procedures, allowing all surgeons to perform thorough lymph node dissections more comfortably and consistently due to enhanced visualization and dexterity. However, although attractive it has not been objectively proven concepts that RATS can enable an “average” surgeon to perform a “master-level” radical dissection with greater ease than VATS. Therefore, it is difficult to foresee when the robotic system will become a vital tool for quality assurance to reduce performance variability across the field.

## Cost effectiveness

The robotic technological advantage comes at a price. The higher cost of RATS compared to VATS remains a significant hurdle to its widespread adoption, raising concerns about cost-effectiveness in healthcare systems (15, 16). Swanson SJ et al. (17) analyzed 15,502 patient records and they found that patient operating times were longer for RATS than VATS (4.49 hours versus. 4.23 hours; P = 0.0959) and wedge resection (3.26 versus. 2.86 hours; P = 0.0003). Length of stay was similar with no differences in adverse events (6, 15–17). The conclusions were that RATS lobectomy and wedge resection seem to have higher hospital costs and longer operating times, without any differences in adverse events (6, 15–17). Furthermore, another study demonstrated higher pain scores in the RATS group compared to the UVATS with 17.1% (RATS) vs 34.3% (UVATS) patients pain free at 14 postoperative day (p = 0.016) (18).

## Tailored surgical strategy

As it is manifest that confusion exists, we advocate for a “Tailored Surgical Strategy.” Surgeons should not feel forced to abandon VATS if they are already achieving excellent oncological results with it. For straightforward cases, VATS remains a cost-effective and proficient approach. Conversely, for anatomically complex cases, post-induction treatments, or when extensive lymphadenectomy is anticipated, the robotic platform could offer an advantage in maintaining surgical quality. Nevertheless, although the association of artificial intelligence with robotic platform could certainly in the future add benefits in surgical practice, we must be certain that these technologies are effective, safe, and available to all. It appears evident that the modern thoracic surgeon must be able to operate using VATS or RATS (19–21) to tailor the operation on the basis of patient’s pathology and surgeon preference; in each case patient’s safety is at the top of mind of all surgeons.

## The future

However, we should welcome RATS not as a replacement for surgical skill, but as a sophisticated instrument that could empowers surgeons to uphold the highest standards of oncological resection. Future discussions should shift from “Which platform is better?” to “How can we use these platforms to ensure every patient receives the same high-quality, standardized surgery?”. Although speculative, we believe that the development of independent humanoid surgical robots could lead to improve patient outcomes reducing the weakness due to “human factors”. Highest standards of oncological resection could be equal worldwide, and also delivered in less developed countries (22).

## Conclusion

In conclusion, in the debate of RATS versus VATS, we must not lose sight of the ultimate goals: the patient’s safety and long-term survival. The literature and our experience suggest that the surgical approach itself is secondary to the quality of the surgery performed, and the surgeon still remains the most important prognostic factor.

## Data Availability

The original contributions presented in the study are included in the article/supplementary material. Further inquiries can be directed to the corresponding author.
